# Mental health outcomes of quarantine and isolation for infection prevention: a systematic umbrella review of the global evidence

**DOI:** 10.4178/epih.e2020038

**Published:** 2020-06-02

**Authors:** Md Mahbub Hossain, Abida Sultana, Neetu Purohit

**Affiliations:** 1 Department of Health Promotion and Community Health Sciences, School of Public Health, Texas A&M University, College Station, TX, USA; 2 Nature Study Society of Bangladesh, Khulna, Bangladesh; 3 The IIHMR University, Jaipur, Rajasthan, India

**Keywords:** Mental health, Mental disorders, Communicable diseases, Quarantine, Systematic review, Meta-analysis

## Abstract

**OBJECTIVES:**

Transmission of infectious diseases is often prevented by quarantine and isolation of the populations at risk. These approaches restrict the mobility, social interactions, and daily activities of the affected individuals. In recent coronavirus disease 2019 (COVID-19) pandemic, quarantine and isolation are being adopted in many contexts, which necessitates an evaluation of global evidence on how such measures impact the mental health outcomes among populations. This umbrella review aimed to synthesize the available evidence on mental health outcomes of quarantine and isolation for preventing infectious diseases.

**METHODS:**

We searched nine major databases and additional sources and included articles if they were systematically conducted reviews, published as peer-reviewed journal articles, and reported mental health outcomes of quarantine or isolation in any population.

**RESULTS:**

Among 1,364 citations, only eight reviews met our criteria. Most of the primary studies in those reviews were conducted in high-income nations and in hospital settings. These articles reported a high burden of mental health problems among patients, informal caregivers, and healthcare providers who experienced quarantine or isolation. Prevalent mental health problems among the affected individuals include depression, anxiety, mood disorders, psychological distress, posttraumatic stress disorder, insomnia, fear, stigmatization, low self-esteem, lack of self-control, and other adverse mental health outcomes.

**CONCLUSIONS:**

This umbrella review found severe mental health problems among individuals and populations who have undergone quarantine and isolation in different contexts. This evidence necessitates multipronged interventions including policy measures for strengthening mental health services globally and promoting psychosocial wellbeing among high-risk populations.

## INTRODUCTION

Quarantine and isolation are public health measures used to prevent the transmission of infectious diseases among individuals and communities [1,2]. Conceptually, quarantine and isolation share the same purpose of infection prevention; however, these terms have distinct meanings in practice. Isolation aims to separate infected individuals from those who have not contracted the infection, whereas quarantine takes a different approach by separating and restricting the movements of people who have been exposed to an infectious disease to monitor whether they develop the disease over time [1].

Historically, quarantine was one of the few known measures to protect lives and cities during the plague epidemics in Europe during the 14th century [3]. Later in the United States, the increasing burden of different infectious diseases, including yellow fever, resulted in the 1878 National Quarantine Act [3,4]. In the past centuries, quarantine became relevant for addressing cholera epidemics and many other historical events related to infectious diseases globally [2,3].

In December 2019, an outbreak of a novel strain of coronavirus occurred in Wuhan, Hubei Province, China, and spread across the world within a short time [5,6]. On February 11, 2020, the World Health Organization (WHO) named it coronavirus disease 2019 (COVID-19) [7]. China implemented a 14-day quarantine for to prevent the transmission of COVID-19 [8]. Nonetheless, the death toll of COVID-19 continued to grow rapidly across the world. With an increasing number of new cases and a high case fatality rate, COVID-19 became a major concern for global health [9]. The WHO acknowledged this crisis and declared COVID-19 a pandemic [10,11]. To address the growing burden of COVID-19, Italy announced a nationwide quarantine [12]. These events brought the attention of the scientific community to quarantine, isolation, and other preventive measures that may protect health and save lives around the world.

Although quarantine and isolation are adopted for protecting individuals’ physical health from infectious diseases, it is also essential to consider the mental health implications of these measures for those who experience such restrictions. People quarantined in earlier outbreaks of infectious diseases have reported adverse mental health outcomes following the quarantine period. A study evaluated the mental health status of 398 parents of children who experienced disease containment and found 30% of the isolated or quarantined children and 25% of the quarantined or isolated parents met the criteria for post-traumatic stress disorder (PTSD) [13]. Another study assessed the mental health status of individuals who were isolated during the Middle East Respiratory Syndrome (MERS) epidemic. This study found that the prevalence of anxiety symptoms and feelings of anger was 7.6% (95% confidence interval [CI], 6.3 to 8.9) and 16.6% (95% CI, 14.8 to 18.4), respectively [14]. A cohort study evaluated the psychological impact of the 2003 Severe Acute Respiratory Syndrome (SARS) outbreak in Canada among 1,912 adults, and found a high burden of psychological distress and symptoms of PTSD (p<0.001) among healthcare providers [15]. Similar studies have provided information on how various mental health conditions may appear when an individual is quarantined or isolated [16,17]. Therefore, evidence on such problems would be useful for informing policy-makers and practitioners about the mental health outcomes associated with quarantine and isolation. Such evidence can facilitate further research and informed decision-making to ensure that the infectious disease or condition is addressed while minimizing the harms to the mental health and wellbeing of the affected individuals.

Evidence synthesis is recognized as a rigorous process wherein the best possible information is identified and critically appraised to inform decision-making in the health sciences [18,19]. As observational or experimental studies may provide a partial understanding of how quarantine and isolation impact human minds, it is essential to combine the findings of multiple primary studies to inform the scientific community and policy-makers through systematic reviews and meta-analyses. This process often becomes more challenging when continued intellectual discourse about a topic results in the development and publication of multiple reviews with similar or conflicting findings. Such differences across studies are acknowledged and analyzed in umbrella reviews or reviews of the reviews [20,21], which aim to find the best possible evidence from existing reviews in a systematic way and to inform evidence-based decision-making.

Since 2015, many umbrella reviews have been conducted to evaluate the evidence base on the psychosocial epidemiology of mental health in diverse populations [22-27]. Although several reviews have reported psychological impacts of quarantine or isolation [28,29], no umbrella review or overview of the reviews was found, although such a review could provide valuable information on the mental health implications of quarantine and isolation within the global landscape. The objective of this umbrella review is to evaluate the mental health outcomes associated with quarantine and isolation from existing reviews. Such evidence may offer broader insights into the psychosocial aftermaths of COVID-19 and empower decision-makers to adopt evidence-based policies to protect individuals’ physical and mental health during and after infectious disease outbreaks.

## MATERIALS AND METHODS

### Guidelines, sources, and processes of collecting the literature

In this umbrella review, we followed the PRISMA (Preferred Reporting Items for Systematic Reviews and Meta-Analyses) guidelines and the recommendations by the Joanna Briggs Institute (JBI) Umbrella Review Methodology Working Group [21,30]. We searched the MEDLINE, Embase, PubMed, Academic Search Ultimate, Health Source: Nursing/Academic Edition, Health Policy Reference Center, American Psychological Association (APA) PsycInfo, Cumulative Index to Nursing and Allied Health Literature (CINAHL), and Web of Science databases using a set of keywords as listed in Table 1.

These keywords were used to capture several domains in the scientific literature. First, quarantine and isolation may be discussed interchangeably in the literature, and different types of isolation have been described in global studies. Several keywords were used to capture this variety of keywords in the existing literature. Second, several keywords were used to identify the literature on infectious diseases, including past outbreaks and the contemporary COVID-19 pandemic. Third, to assess the global literature in an inclusive manner, we adopted a broader definition of mental health in this review. We considered any mental disorders listed in the International Classification of Diseases or Diagnostic and Statistical Manual of Mental Disorders, which include depression, anxiety, substance and alcohol use disorders, sleep disorders, and other psychiatric conditions [31,32]. We also included psychological and behavioral conditions, including but not limited to self-esteem, loneliness, and psychological distress, that are integral to mental health and wellbeing [33,34]. The inclusion of conditions was consistent with the WHO definition of health [35], which motivated this review to include broader outcomes and determinants associated with mental health alongside evaluations of mental disorders. Lastly, we used keywords for including systematically conducted reviews with different names. A review reported the existence of at least 14 types of reviews [36], which informed our choice of keywords to identify all review articles that had a systematic methodology of searching the literature for the respective review question. We combined these keywords with appropriate Boolean operators (OR/AND) and searched within the titles, abstracts, subject heading (e.g., Medical Subject Headings [MeSH]), and other search fields. Moreover, we performed manual searching of the reference lists of selected articles, published studies that were highly cited in the field, and newer articles that cited the earlier articles. This manual searching was conducted in the Google Scholar database. Furthermore, we reached out to subject matter experts to identify potential studies that may have met our criteria. The entire search process was conducted since the inception of the respective databases and was updated until March 10, 2020.

### Inclusion and exclusion criteria

We included an article in this umbrella review if it fulfilled all the following inclusion criteria: (1) it was published in a peer-reviewed journal, (2) the language of the full-text article was English, (3) it was a review article with a clearly stated methodology of searching the literature (for example, systematic reviews, meta-analyses, systematic scoping reviews, etc.), (4) it reported any mental health-related conditions (for example, mental disorders such as PTSD or mental health conditions such as fear or loneliness), (5) the participants of the primary studies in the respective reviews had experienced quarantine or any form of isolation for infection prevention in any capacity (for example, patients, their informal caregivers, or healthcare providers who were involved in the quarantine or isolation process), (6) populations from any socio-demographic background or participants with known medical conditions were included (for example, children, adults, elderly, or individuals with any diseases or infections were included), and (7) it was published at any time within the search period. Lastly, we excluded articles that did not meet at least one of the above-mentioned criteria.

### Screening and selection of the literature

All the citations found through searching the databases and additional sources were uploaded to RefWorks [37], which was used to manage the citations data and to exclude duplicate citations from the total collection of literature. Further, these citations were exported to Rayyan [38], which is a cloud-based platform for screening citations data. Two authors (MMH and AS) independently screened all the citations according to the inclusion and exclusion criteria of this review. At the end of the primary screening, any discrepancies during the screening process were resolved based on a discussion in the presence of the third author (NP). Then, the full-texts of the preliminarily selected articles were reviewed to evaluate their eligibility for this review and excluded if they did not meet all criteria as stated earlier.

### Data extraction and analysis

We extracted data from the finally selected articles using a manual data extraction form. Two authors independently extracted data on the following domains: titles and objectives of the reviews, the number of databases searched, the timeframe of conducting the search process, types of the primary studies included in the reviews, the countries of origin of those studies, sample sizes, characteristics of the study participants, the infectious conditions or agents that were the primary reasons for quarantine or isolation in the respective studies, and the mental health outcomes reported in the reviews. A narrative synthesis was conducted due to heterogeneity in methods, population characteristics, reasons for quarantine or isolation, and mental health outcomes in the respective reviews.

### Quality assessment

We used the JBI critical appraisal checklist for systematic reviews and research synthesis [21] to assess the methodological quality of studies included in this umbrella review. This checklist consists of 10 items dealing with different methodological aspects of a review article, including the appropriateness of the search strategies, the approach to synthesizing evidence, potential sources of biases, and prospects for future research and policy-making. In this review, 2 authors independently evaluated each of the included articles. On this 10-item checklist, each item can receive 1 point, and the overall quality score of a study can range from 0 to 10. In this umbrella review, studies receiving 0-4, 5-7, and 8-10 points were categorized as low-quality, medium-quality, and high-quality studies, respectively. The scoring and categorizing processes in this review were informed by earlier umbrella reviews [26,27,39].

### Ethics statement

No ethical approval was required as this is a systematic review of published reviews and it did not involve any human participants.

## RESULTS

### Characteristics of the included articles

**Figure 1.**
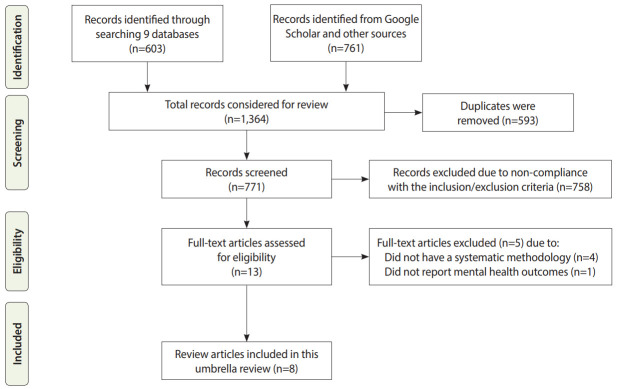
Flow diagram of the literature search process.

We found a total of 603 citations from MEDLINE (n=128), Embase (n=114), PubMed (n=131), Academic Search Ultimate (n=43), Health Source: Nursing/Academic Edition (n=16), Health Policy Reference Center (n=2), APA PsycInfo (n=17), CINAHL (n=48), and Web of Science (n=104). In addition, Google Scholar and additional sources provided 761 citations. In total, 1,364 citations were considered in this review and 771 unique records were screened after removing 593 duplicate records (Figure 1). After full-text screening, 8 reviews were included in this umbrella review (Table 2) [28,29,40-45]. These reviews were published between 2009 and 2020, and most reviews (n=5) were published since 2018. The reviews used different scholarly sources, ranging from 2 to 4 databases. The number of primary studies in those reviews ranged from 7 to 26. Most reviews included cohort studies (n, 6: number/range of primary studies in each review; S, 1 to 12), followed by cross-sectional studies (n, 5; s, 2 to 11), qualitative studies (n, 3; s, 2 to 10), case-control studies (n, 1; s, 6), quasi-experimental studies (n, 2; s, 2), case studies (n, 1; s, 2), mixed-method studies (n, 1; s, 2), reviews (n, 1; s, 1), and psychological evaluations (n, 1; s, 1). In the quality assessment (Supplementary Material 1), 3 reviews were found to have high quality [28,44,45], while 5 had medium quality [29,40-43].

### Characteristics of the study populations

The reviews included primary studies ranging from case studies with 1 subject to larger samples (e.g., 9,648). Three reviews did not specify the origin of the primary studies [40,41,43]; among the remaining reviews, most of the primary studies were from the United States, United Kingdom, and Canada, whereas fewer studies were conducted in Sweden, Australia, Netherlands, Korea, Senegal, New Zealand, Ireland, Brazil, Liberia, Turkey, France, Spain, Sierra Leone, Hong Kong, Taiwan, China, and Singapore [28,29,42,44,45]. Most reviews included primary studies conducted in healthcare settings. For example, Gammon et al. [28] reviewed 14 studies with samples ranging from 1 to 528, whereas Purssell et al. [44] reviewed 26 studies with samples ranging from 14 to 9,684. Both reviews evaluated studies that recruited participants from clinical settings, including healthcare providers and clinical students. In contrast, a review by Brooks et al. [29] included studies that recruited participants, including patients, providers, students, institutional stakeholders, and community members from diverse settings.

### Infectious diseases or conditions for quarantine and isolation

Different types of measures for infection prevention and associated causes were reported across reviews (Table 3). Abad et al. [41] evaluated studies focusing on isolation, whereas 3 studies specified source isolation in the primary studies [28,42,43]. Moreover, 3 reviews focused on contact precaution or isolation [40,44,45]. One study by Brooks et al. [29] emphasized primary studies conducted on quarantine.

Across the study populations, quarantine or isolation measures were taken in response to several infectious agents or conditions. Methicillin-resistant *Staphylococcus aureus* (MRSA) was the most commonly reported (number of reviews, 6) reason for isolating the patients [28,41-45]. Four reviews reported multi-drug resistant organisms as the primary reason for isolation [40,41,44,45]. Several reviews reported that SARS (n=3) and vancomycin-resistant *Enterococcus* (n=2) were the reasons for isolation [29,41,42]. Other infectious agents or conditions associated with isolation or quarantine included healthcare-associated infections, tuberculosis, Ebola, H1N1 influenza, equine influenza, and MERS [29,41,43].

### Mental health outcomes of quarantine and isolation

The reviews reported a high burden of mental health conditions among individuals who experienced isolation or quarantine [28,29,45]. For example, Gammon et al. [28] found that 33% of the participants who had undergone source isolation had poor mental health status. Among specific mental health outcomes, all reviews reported a high prevalence of anxiety among study participants [28,29,40-45]. For example, Purssell et al. [44] found that the pooled standardized mean difference for anxiety was 1.45 (95% CI, 0.56 to 2.34) among participants who experienced contact precautions and isolation.

Six reviews reported varying levels of depression among the study participants [29,41-45]. For example, Sharma et al. [45] found pooled mean difference estimates for the Hospital Anxiety and Depression Scale to be -1.85 (p=0.09), whereas Purssell et al. [44] found the pooled mean difference to be 1.28 (95% CI, 0.47 to 2.09) for depression among the study participants. Four reviews reported anger and irritability among the study participants [29,40-42]. For example, a review found that up to 57% of the participants reported irritability alongside other mental conditions following the quarantine [29]. Psychological distress associated with suboptimal patient-provider communication was reported in 4 reviews [28,40,41,43]. Moreover, 4 reviews found varying levels of stress among the study participants who experienced quarantine or isolation [28,29,42,43].

Several psychosocial conditions affected the mental health and wellbeing of the individuals during and after quarantine or isolation. Three reviews found that the participants perceived social exclusion or felt neglected [40,42,43]. Often, psychological and emotional disturbances were reported by the affected individuals, as found in 3 reviews [29,42,43]. Stigmatization was reported in 3 reviews, which impacted the study participants’ mental health and wellbeing [28,42,43]. For example, Gammon et al. [28] found that 32% of MRSA carriers reported stigma, among which 14% of the participants reported “clear stigma” and 42% reported “suggestive for stigma.”

Quarantine and isolation for infection prevention also impacted the mental health and wellbeing of healthcare providers [28,29]. For example, Brooks et al. [29] found several mental health conditions among the healthcare providers who worked under quarantine, including acute stress disorder, exhaustion, detachment, anxiety, depression, irritability, insomnia, poor concentration, deterioration of work performance, alcohol use, avoidance behavior, and posttraumatic stress-related symptoms, even 3 years after the quarantine period. Moreover, the mental health of informal caregivers was affected due to quarantine and isolation. Brooks et al. [29] reported that 28% of parents of children who were quarantined had trauma-related mental disorders, which was higher than comparison parents who had a prevalence of 6% for the same condition.

Several other mental disorders and psychological conditions were found across study populations, including low self-esteem [40,41,43], mood disorders [29,43], fear [29,41], guilt [29], loneliness [28,41-43], boredom [41,42], feeling a lack of control [28,42,43], insomnia [29], PTSD [29], perceived dirtiness [43], vigilant handwashing [29], and avoiding crowds and social gatherings even after quarantine or isolation [29]. One study in the review by Abad et al. [41] reported that a few participants acknowledged positive feelings of privacy and freedom during isolation, whereas the remaining studies reported higher scores for depression, anxiety, anger-hostility, fear, loneliness, boredom, and low self-esteem.

## DISCUSSION

To the best of our knowledge, this is the first umbrella review to evaluate the global evidence on mental health outcomes associated with quarantine and isolation measures for infection prevention. Most reviews included cohort studies as well as qualitative studies, which enabled them to explore how periods of restricted mobility not only addressed the transmission of infectious diseases, but affected the mental health and wellbeing of the study participants. Some of the reviews found that the impacts of quarantine and isolation continued over a longer period, highlighting how acute exposure to psychosocial stressors during quarantine and isolation can exert prolonged impacts on the human mind, psychological processes, and mental health outcomes. Such effects were found among patients, informal caregivers, and healthcare providers, indicating that complex psychosocial dynamics take place among the key stakeholders in the process of quarantine or isolation who are likely to be affected and to experience negative mental health outcomes. These findings were consistent across most reviews and primary studies included in the respective reviews. Moreover, most reviews included in this umbrella review had medium quality and 3 reviews had high quality, whereas no review was found to have low quality. As the included reviews were heterogeneous in their methods, populations, and outcomes, no conclusion can be drawn on how the quality of the reviews could have mediated the comparative findings of the respective reviews. However, as none of the reviews received a low score for quality in the assessment process, this umbrella review found consistency in the findings of the analyzed reviews. Quarantine and isolation impacted mental health and wellbeing across populations in different contexts, and this finding remains a critical concern for global health discourse. However, several issues should be considered to further evaluate these findings and to draw meaningful insights for future research, policy-making, and practice.

First, most studies in the included reviews originated from high-income countries, which may affect the generalizability of the findings to low-income and middle-income countries (LMICs). These countries are often under-represented in terms of generating evidence through empirical studies [46], which remains a major concern for strengthening the global evidence base on psychosocial epidemiology. Therefore, this review underscores the need to conduct more studies in LMICs to better understand how quarantine or isolation may affect mental health and wellbeing in those contexts.

Second, patients and their informal caregivers experienced a high burden of mental disorders, which necessitates integrating psychosocial care and mental health support alongside physical health services during quarantine or isolation for infection control. Existing models of care may need human contact to deliver such services. However, recent advancements in digital health interventions may address such issues and facilitate delivering mental health interventions using digital platforms with minimal human involvement [47-49]. Future research and implementation strategies should explore such avenues to improve mental health outcomes during infectious disease outbreaks.

Third, healthcare providers have reported experiencing various mental health problems, including emotional exhaustion, which may result in suboptimal performance at the workplace, as found in this umbrella review. Several evidence-based reviews have reported a high burden of professional burnout among healthcare providers [50-52], which may be exacerbated during quarantine and isolation for infection prevention. Such evidence suggests the need for academic and professional approaches to sensitize clinical students and healthcare providers to be aware of such issues in practical settings and to adopt protective mental health measures before working in such stressful conditions. Moreover, evidence-based psychosocial interventions for improving mental health and wellbeing among healthcare providers should be adopted [53].

Fourth, most of the reviews synthesized evidence from populations in clinical settings. This highlights the significance of healthcare organizations during isolation and quarantine. Such examples have become evident during the COVID-19 pandemic; for instance, healthcare organizations in China played critical roles in treating infected individuals and preventing the outbreak within their scope [54]. It is necessary to revisit existing protocols and resources in health services organizations so that their preparedness for providing mental healthcare in quarantine and isolation can be ensured.

Fifth, the profile of infectious conditions that were associated with quarantine and isolation in this review involved a variety of agents, limiting the degree to which conclusions can be drawn on how different agents may have required different levels of isolation or impacted mental health among the participants differently. Moreover, scarce insights relevant for the COVID-19 pandemic can be drawn from previous studies analyzing different conditions. Furthermore, the global research trends on COVID-19 have not adequately explored the psychosocial impacts of this ongoing crisis [55], which underscores the critical need for more research in this domain. However, studies on SARS and MERS outbreaks provide some insights on how coronaviruses have affected mental health in earlier outbreaks. The current evidence base should be considered when designing future studies and interventions for COVID-19 and other infectious conditions.

Sixth, the current evidence provides information on different mental health problems associated with quarantine and isolation, which may also need psychosocial perspectives to assess the way these preventive measures are enforced globally. Rather than mandating such approaches, altruistic social behavior and practices should be promoted [29]. Moreover, the early engagement of infected individuals, caregivers, or populations at risk may allow all parties to make informed decisions and to address anxiety and distress related to uncertainty about potential risks and benefits [56,57].

Seventh, interpersonal relationships, networks, and social capital appear to have critical significance during major health events, including quarantine and isolation [58]. Such ties must be explored and leveraged to improve mental health outcomes during infection prevention. For example, a study reported a few participants who acknowledged higher levels of privacy and freedom during isolation [41]. This highlights how perceptions can be different and how individual ideas and perceived stressors may result in diverse mental health outcomes. Therefore, individual psychosocial factors should be thoroughly evaluated to identify risk and protective factors among individuals, which may guide the development and adoption of personalized mental health measures. Other opportunities to strengthen mental healthcare may include interventions for improving patient-provider communication, social media interventions, online support groups, and other resources appropriate to the contexts and psychosocial preferences of the affected individuals.

Eighth, awareness is one of the key determinants of mental health among individuals and populations [59]. It is essential to acknowledge the role of knowledge and attitudes about mental health, especially during quarantine and isolation, which may reduce stigmatization as well as promote resilience to psychosocial problems. The presence of co-occurring physical or mental health problems may exacerbate the psychological challenges during quarantine and isolation. It is recommended that infection control measures should be included in existing health promotion programs so that psychosocial preparedness can be developed at the population level, which may profoundly help during unforeseen infectious crises.

Ninth, the effectiveness of isolation or quarantine may depend on the structure and functions of different organograms in a health system. Although measures often focus on crude indicators such as the incidence or mortality rate, little is known about how the levels of preparedness of health systems contribute to assure the citizens during outbreaks of major infectious diseases. This may impact the way an outbreak or potential infection is perceived by people across societies. The ongoing COVID-19 pandemic has resulted in diverse responses from health systems in different countries. The current review found varying levels of mental health outcomes globally, which necessitates strengthening health systems’ capacities to improve mental health among the affected populations. Moreover, future research is necessary to understand how different health systems react to small-scale to large-scale outbreaks, and how such responses influence mental health status across populations.

Last but not least, infection prevention requires stricter measures to standardize the processes and ensure the quality of such services globally. During large-scale crises like COVID-19, this need is perceived strongly throughout the international scientific community, which has been reflected in extensive collaborative research since the start of the COVID-19 outbreak [55]. However, global mental health remains a developing domain in health sciences, meaning that little information is available on how global institutions and stakeholders can contribute together to improve mental health outcomes among diverse population groups around the world. It is necessary to develop a global alliance, perhaps an institution under the leadership of major global health stakeholders, which may work on improving global mental health with a focus on providing support to regional and local institutions for building capacities and resources for mental health. Such efforts may create and strengthen mental health support networks, allowing timely actions to respond to infectious conditions, to promote psychosocial resilience, and to protect mental health among individuals and populations simultaneously.

This review has several limitations, which must be acknowledged. We did not include articles beyond the strategy outlined in this review. This may have resulted in selection bias as there are many more databases with potential studies that could have met our criteria. Another limitation is publication bias, which may have limited the synthesis of evidence from unpublished studies. Moreover, the heterogeneous methods and outcomes of the reviews included in this study do not provide insights on how different levels of quarantine or isolation may have had distinct impacts on mental health outcomes in different contexts, which remains a limitation of this review. Last but not least, an umbrella review evaluates reviews, rather than synthesizing study-level evidence [21]; such meta-epidemiological analyses may have different objectives or outcomes, which were beyond the scope of this review. These limitations should be considered in translating the evidence of this review into practice and conducting future research in this area.

## CONCLUSION

This umbrella review synthesized the global evidence on mental health outcomes of quarantine and isolation for infection prevention. The current evidence informs a high burden of different mental health problems among patients, informal caregivers, and healthcare providers. These challenges must be recognized for strengthening mental health services during quarantine and isolation. Moreover, risk and protective factors of mental health among individuals and populations should be evaluated to inform the future development and implementation of multilevel interventions aiming to ensure optimal mental health and wellbeing when individuals experience complex psychosocial stressors due to restricted mobility and social interactions. Lastly, humane caregiving should be placed at the center of infection control, ensuring scientific standards to achieve collective goals in protecting physical and mental health among populations at risk.

## Figures and Tables

**Table 1.** Keywords used for searching databases

Search query	Keywords (searched within titles, abstracts, subject headings such as MeSH, and general keywords)
1	“quarantine” OR “isolation” OR “source isolation” OR “contact isolation” OR “patient isolation” OR “confinement”
2	“infection” OR “infected” OR “infective” or “infectious” or “communicable” OR “COVID” OR “COVID-19” OR “nCoV” OR “corona-virus” OR “MERS” OR “SARS” OR “outbreak” OR “epidemic” OR “pandemic”
3	“mental health” OR “mental disorders” OR “mental illness” OR “psychiatric” OR “psychological” OR “psychosocial” OR “adverse outcomes” OR “unintended consequences” OR “depression” OR “depressive” OR “sleep disorder” OR “insomnia” OR “anxiety” OR “PTSD” OR “suicide” OR “self-harm” OR “suicidal” OR “distress” OR “affective” OR “fear” OR “phobia”
4	“systematic review” OR “systematic literature review” OR “evidence-based review” OR “meta-analysis” OR “meta-analytic” OR “meta-regression” OR “pooled effect” OR “pooled estimate” OR “scoping review” OR “rapid review” OR “evidence-based practice” OR “systematized review” OR “literature review” OR “review of the literature”
Final search query	1 AND 2 AND 3 AND 4

MeSH, Medical Subject Headings.

**Table 2.** Characteristics of the articles included in this review

Study	Name and timeframe of databases searched	Name and timeframe of databases searched	Country or locations of the primary studies	Quality of the review	Sample size and characteristics
Morgan et al., 2009 [40]	MEDLINE, PubMed, Google Scholar, and additional sources; 1970-2008	7 studies on mental health outcomes: 5 cohort studies, 2 cross-sectional and series interviews	Not specified	Medium	Sample size ranged from 8 to 43; participants in 7 selected studies; most (n=6) studies recruited hospitalized patient populations, 1 study included both patients and providers
Abad et al., 2010 [41]	MEDLINE and CINAHL; 1966-2009	8 cohort studies and 7 case-control studies	Not specified	Medium	Sample size ranged from 16 to 156; most studies had adult participants; 2 studies recruited children; samples were recruited from hospital wards
Barratt et al., 2011 [42]	MEDLINE, CINAHL, PsycINFO, and Cochrane Library Databases; 1990-2010	Studies were qualitative (n=7), cohort (n=7), cross-sectional (n=6), case studies (n=2), and review (n=1)	Most studies were from the UK (n=6) followed by the US (n=4), Hong Kong (n=1), and Canada (n=1)	Medium	Sample size ranged from 7 to 300; samples were recruited from different clinical settings
Gammon et al., 2018 [43]	PubMed and ASSIA; 1990-2017	Not specified	Not specified	Medium	Sample size ranged from 13 to 41 among studies reporting sample sizes; participants were recruited from different hospital wards
Gammon et al., 2019 [28]	MEDLINE and ASSIA; 1990-2017	14: only 1 study was cohort-based; most studies were cross-sectional, and 10 studies had a qualitative design	Most studies were from the UK (n=6), followed by the US (n=2), Sweden (n=2), and 1 study each from the Netherlands, New Zealand, Ireland, and Brazil	High	Sample size ranged from 1 to 528; most studies recruited patients and providers from clinical settings, whereas 2 samples included nursing students
Brooks et al., 2020 [29]	MEDLINE, PsycINFO, Web of Science; timeframe not specified	25: cross-sectional (n=11), qualitative (n=7), longitudinal (n=1), observational (n=2), mixed methods (n=3), and psychological evaluation (n=1)	Most studies were conducted in Canada (n=8) and China (n=4); 2 studies each from Taiwan, Australia, Korea, and Liberia; 1 study each from Sierra Leone, Senegal, Hong Kong, and Sweden; 1 study had participants both from the US and Canada	Medium	Sample size ranged from 10 to 6,231; diverse samples including patients, providers, students, institutional stakeholders, and community members were recruited
Purssell et al., 2020 [44]	Embase, MEDLINE, and PsycINFO; from the inception of the databases until December 2018	26: cohort (n=12), case-control (n=6), cross-sectional (n=4), and quasi-experimental (n=2) studies	Most studies were from the US (n=14), followed by the UK (n=3), Canada (n=3), and 1 study each from Spain, Turkey, Netherlands, Singapore, France, and 1 study had participants both from the US and Canada	High	Sample size ranged from 14 to 9,684; patients were recruited from diverse clinical settings
Sharma et al., 2020 [45]	Embase, PubMed, and Google Scholar; studies published through March 2019	7: cohort (n=4), quasi-experimental (n=2), and not specified (n=1)	Not specified	High	Sample size ranged from 16 to 148; participants were recruited from diverse clinical settings

CINAHL, Cumulative Index to Nursing and Allied Health Literature; ASSIA, Applied Social Sciences Index and Abstracts.

**Table 3.** Mental health outcomes in different conditions of quarantine and isolation

Study	Type and reasons for quarantine, isolation, or other measures for infection prevention	Mental health impacts
Morgan et al., 2009 [40]	Contact precaution; MDROs	Patients expressed feeling neglected, isolated, angry (p=0.037), depression (up to 77%, p-values ranged from < 0.01 to < 0.001), anxiety (p<0.001), low self-esteem (p<0.01), perception of less control (p<0.001); less patient-provider contact was reported
Abad et al., 2010 [41]	Isolation; multiple infectious conditions including VRE, MRSA, healthcare-associated infections, MDRO, SARS, and mixed infections	Most studies reported higher scores for depression, anxiety, anger-hostility, fear, loneliness, boredom, and low self-esteem; One study reported higher freedom and privacy perceived by the patients; higher anxiety scores were associated with history of mental illness; Most studies found that providers visited less frequently and spent less time with isolated patients compared to the controls
Barratt et al., 2011 [42]	Source isolation; VRE, MRSA, SARS, and mixed infections	Studies reported stress, anxiety, depression, loneliness, anger, neglect, abandonment, boredom, stigmatization, low sense of control and self-esteem, and negative emotions
Gammon et al., 2018 [43]	Source isolation; MRSA, tuberculosis, and other non-specified infections	Participants experienced limited visiting, lack of attention and lesser interaction with providers, and disruption of routine; Additionally, feelings of loneliness, abandonment, social exclusion, stigmatization, anxiety, depression, mood changes, stress, negative effects on coping and psychological functioning, low self-esteem and sense of control, emotional problems, anger, perceived feeling of dirtiness, and a lack of clarity on the isolation process were reported; Moreover, studies have found that many psychosocial issues were attributable to the primary cause(s) of hospitalization
Gammon et al., 2019 [28]	Source isolation; MRSA and other non-specified infectious conditions	Patients reported a lack of control and feeling lonely in isolation, which led to a perceived state of social exclusion; Along with poor mental health (33%), about 32% of MRSA carriers reported stigma; of these, 14% reported “clear stigma” and 42% reported “suggestive for stigma”; Patients also reported suboptimal patient-provider communication, lack of understanding facial expression due to masks, and procedures that provoked anxiety and stresses of isolation
Brooks et al., 2020 [29]	Quarantine; SARS (n=15), Ebola (n=5), H1N1 influenza (n=3), Middle East Respiratory Syndrome (n=2), and equine influenza (n=1)	Patients reported general psychological problems, emotional disturbance, depression, stress, low mood (up to 73%), irritability (up to 57%), anger, guilt, nervousness, sadness, fear, numbness, vigilant handwashing and avoidance of crowds even after quarantine period; The parents and children who were quarantined had higher prevalence of trauma-related mental disorders (28% parents had such symptoms compared to 6% control parents); Healthcare providers also reported acute stress disorder, exhaustion, detachment, anxiety, depression, irritability, insomnia, poor concentration, deterioration of work performance, alcohol use, avoidance behavior, and posttraumatic stress-related symptoms even 3 yr after the quarantine period
Purssell et al., 2020 [44]	Contact precaution and isolation; MRSA and MDROs	The pooled standardized mean difference was 1.28 (95% CI, 0.47 to 2.09) for depression and 1.45 (95% CI, 0.56 to 2.34) for anxiety among the study participants
Sharma et al., 2020 [45]	Isolation precaution; MRSA, MDROs, and other infections	The pooled mean difference estimates for HADS-A was -1.4 (p=0.15) and that for HADS-D was -1.85 (p= 0.09) for anxiety and depression, respectively; Most studies (n=6) reported negative effects on psychological burden scales in the empirical analysis

MDROs, multiple drug-resistant organisms; VRE, vancomycin-resistant Enterococcus; MRSA, methicillin-resistant *Staphylococcus aureus*; SARS, Severe Acute Respiratory Syndrome; CI, confidence interval; HADS, Hospital Anxiety and Depression Scales.
