# Supplementary material for: Mental health outcomes of quarantine and isolation for infection prevention: a systematic umbrella review of the global evidence
Source: Epidemiol Health. 2020 Jun 2;42:e2020038. doi: 10.4178/epih.e2020038 (PMC7644933; doi:10.4178/epih.e2020038)
Supplement: Supplementary Material 1. [file epih-42-e2020038-suppl.pdf]

Volume: 42, Article ID: e2020038

https://doi.org/10.4178/epih.e2020038

Supplementary Material 1: Critical appraisal of the quality of the included reviews

Authors/ Sources	Is the review question clearly and explicitly stated?	Were the inclusion criteria appropriate for the review question?	Was the search strategy appropriate?	Were the sources and resources used to search for studies adequate?	Were the criteria for appraising studies appropriate?	Was critical appraisal conducted by two or more reviewers independently?	Were the methods used to combine studies appropriate?	Was the likelihood of publication bias assessed?	Were recommendations for policy and/or practice supported by the reported data?	Were the specific directives for new research appropriate?	Overall score and quality rating
Morgan et al. (2009) [40]	Yes	Yes	CD	Yes	CD	CD	Yes	No	Yes	Yes	6 (Medium)
Abad et al. (2010) [41]	Yes	Yes	Yes	Yes	CD	Yes	Yes	No	CD	Yes	7 (Medium)
Barratt et al. (2011) [42]	Yes	CD	Yes	Yes	No	CD	Yes	No	Yes	No	5 (Medium)
Gammon and Hunt (2018) [43]	Yes	Yes	Yes	CD	No	CD	Yes	No	Yes	Yes	6 (Medium)
Gammon et al. (2019) [28]	Yes	Yes	Yes	Yes	Yes	CD	Yes	No	Yes	Yes	8 (High)
Brooks et al. (2020) [29]	Yes	Yes	Yes	CD	No	CD	Yes	No	Yes	Yes	6 (Medium)
Purssell et al. (2020) [44]	Yes	Yes	Yes	Yes	Yes	CD	Yes	No	Yes	Yes	8 (High)
Sharma et al. (2020) [45]	Yes	Yes	Yes	Yes	CD	Yes	Yes	No	Yes	Yes	8 (High)

(Abbreviations: CD= could not determine)
